# LONGEVITY OVER THE COUNTER: INVESTIGATING THE EFFECTS OF COMMON DIETARY SUPPLEMENTS ON AGING

**DOI:** 10.1093/geroni/igz038.384

**Published:** 2019-11-08

**Authors:** Ben Blue, Elena Vayndorf, Matt Kaeberlein

**Affiliations:** University of Washington, Seattle, Washington, United States

## Abstract

C. elegans has been a workhorse within the field of aging biology for several decades due to its short lifespan, easy culturing, and robust genetic tools. However, the limiting factor in using C. elegans has been that throughput was constrained by the time and effort needed to manually check the worms for signs of life during longitudinal studies. By using the WormBot, a robotic image capture platform, we are able to successfully screen a wide array of compounds for their effects upon C. elegans lifespan. A single WormBot can monitor 144 individual experiments simultaneously and allows for accurate time of death calls. Here we present data generated with the WormBot that includes a screen of compounds from a wide array of natural and synthetic products that are often available as over-the-counter supplements. In order to better examine the effects of these widely-used compounds upon the aging process and an age-associated disease we examined longevity in a wildtype strain of C. elegans as well as an engineered strain that expresses human Aβ protein in the body wall muscle. The age-related pathogenesis of the Aβ-expressing strain is a progressive paralysis that can be halted with treatment of known effectors of Alzheimer’s disease. As such, we screened our battery of compounds with this strain to determine which compounds have a significant affect on delaying Aβ-associated paralysis. Lastly, using the WormBot’s ability to capture video recording, we examine how each compound affects mobility as animals age.

